# Workers’ Self-management, Recovered Companies and the Sociology of Work

**DOI:** 10.1177/0038038514539064

**Published:** 2014-10

**Authors:** Daniel Ozarow, Richard Croucher

**Affiliations:** Middlesex University, UK; Middlesex University, UK

**Keywords:** Argentina, cooperatives, labour, Marxism, social movements, worker-recovered companies, workers’ self-management

## Abstract

We analyse how far Argentina’s worker-recovered companies (WRCs) have sustained themselves and their principles of equity and workers’ self-management since becoming widespread following the country’s 2001–2 economic crisis. Specialist Spanish-language sources, survey data and documents are analysed through four key sociological themes. We find that the number of WRCs has increased in Argentina, and that they represent a viable production model. Further, they have generally maintained their central principles and even flourished. This occurred despite the global economic crisis, legal and financial pressures to adopt capitalist practices and management structures, the risk of market absorption and state attempts to coopt, demobilise and depoliticise the movement. We argue that today they function as a much-needed international beacon of an alternative vision for labour and that integration of their experience has potential to revitalise the field.

## Introduction

This article analyses how far Argentina’s worker-recovered companies (WRCs) have managed to become sustainable production models whilst maintaining their values of equity and workers’ self-management. We illustrate how this response to the global crisis offers viable channels for reinvigorating the sociology of work.

The long-standing experience of worker cooperatives has strong resonance internationally and the Argentine movement, which became a widespread phenomenon a decade ago, has attracted particular attention from academics and labour activists. Given its potential as an alternative model, Argentina’s WRCs act both as beacons and as laboratories for the idea of workers’ emancipation and have therefore aroused interest among Marxists including ourselves. Researchers have however identified market, legal and financial pressures, as well as state attempts to coopt, demobilise and depoliticise the movement (Dinerstein, 2007; Sitrin, 2012; Upchurch et al., 2014). Nevertheless, Argentine WRCs generally survive and largely maintain their central values of equity and worker self-management.

Although Argentina’s experiment with workers’ self-management is perhaps the most widespread and sustained of national experiences in recent times, there is a dearth of pre-crisis English-language material that analyses the experience. This may be due to North American and European assumptions that the proliferation of such models would be confined to the global south. However, recent evidence suggests that cooperative workplaces have been particularly resilient during Europe’s economic crisis (CECOP, 2013) and a number of WRCs like Vio.me in Thessaloniki and Ri-Maflow recycling plant in Milan have emerged. In a manifestation of ‘resonance’ (Holloway, 2010: 77), their workers have been inspired by the Argentine example, generating renewed interest among European and North American audiences in the notion that workers can take over and successfully operate businesses themselves. Indeed, early 2014 witnessed the first exchange between European and Latin American recovered companies when 200 activists, researchers and workers from WRCs across the two continents gathered at the Fralib herb processing and packaging factory near Marseille.

The Argentine case demonstrates that workers’ self-management can flourish in a 21st-century capitalist economy and influence state policy (Dinerstein, 2007), worker subjectivities (Bialakowsky et al., 2005; Coraggio and Arroyo, 2009; Fernández et al., 2008; Monteagudo, 2008) and the politics of wealth distribution and social relations (Flores, 2002; Sitrin, 2012). This alternative ‘moral economy’ – as the humanistic Marxist Edward Thompson (1970) first conceptualised it – has potential to re-inspire the sociological imagination through expanding perceptions of industrial and social possibility.

WRCs have specific characteristics in relation to other forms of ‘social enterprise’ or workers’ cooperatives. Producer cooperatives have a long history internationally, but remain the labour movement’s least well-documented arm. ‘Workers’ cooperatives’ constitute a narrower sub-set; they are run by workers, not external groups that concern themselves with ends other than workers’ welfare. From a classical neoliberal economic viewpoint, their entire rationale is ‘welfare production’ rather than serving the ends of external owners. WRCs are an even narrower sub-category of workers’ cooperatives: here, workers have taken over previously existing businesses that have gone bankrupt and ceased activity, to then recommence production in the absence of the old owner. They seek to enact principles of equity, a value that is reflected in their internal democratic institutions and horizontal organising structures (Sitrin, 2012).

Argentine WRCs’ ideal is of comprehensive control over production, frequently associated with conceptions of societal transformation (MNER, 2003). We apply the ‘self-management’ term to them, rather than ‘workers’ control’ since the former is a more encompassing definition. ‘Industrial democracy’ is another cognate term but Batstone et al. (1979) argue that it is a profoundly problematic concept, and that its quality cannot be established simply by the existence of representative institutions. Political leaderships emerge in workers’ organisations, and impact how ‘democracy’ is interpreted and enacted. Although Pizzi and Icart (2014) show that certain links exist between the scope of democratic practice and equality they offer, and the degree of accompanying social mobilisation in WRCs, more research is required on precisely how these processes have played out.

The proportion of the 13,400 workers involved in Argentina’s estimated 309 WRCs (which are concentrated in small and medium-sized enterprises – SMEs) is just 0.1 per cent of its formally employed population (Programa Facultad Abierta, 2013). Yet they derive importance from their considerable influence on Argentine politics, their transnational symbolic significance, their longevity and, we argue, the implications they carry for the sociology of work.

We propose that resistance to the crisis through workers’ self-management potentially redefines the boundaries of the sociology of work at two distinct levels. First, *practically*, because following an evaluation of their viability, they continue to propagate a credible alternative vision of industrial organisation. Second, *theoretically*, as an examination of this model provides a pathway to reconnect the discipline to broader social theory and to industrial relations debates.

We continue by tracing how sociologists have interpreted the cooperative experiments of the 1970s and more recent developments within the industrial sociology and industrial relations literature. Although these are complex fields, we then show how research on one of their strands – workers’ self-management – can be located within the sociology of work and we propose ways of reconnecting it to mainstream social theory using key themes suggested by Halford and Strangleman (2009). How we apply these authors’ criteria to assess the WRC movement’s achievements and weaknesses is subsequently outlined in the description of our methods. The Argentine case study is then introduced and an analysis of whether they have maintained their ideals is conducted using these themes to shed light on the possibilities that this production model offers as a viable alternative vision of work. We conclude by reflecting on our findings and arguing for their wider significance.

## Workers’ Self-management and Sociological Debates

Until the early 2000s, the most recent wave of workers’ self-management occurred during the 1970s when numerous factory occupations occurred in Italy, France, Portugal and the UK (Ness and Azzellini, 2011). The potential for it to have a transformative impact on society was a central theme of industrial sociology.

In Britain, debates developed in Marxist industrial sociology about whether the creation and proliferation of workers’ cooperatives could provide the basis for socialist transformations or, as Luxemburg (1986[1900]) claimed, were incapable of surviving permanently when operating within capitalist constraints. Hyman (1974) and Miliband (1969) argued along such lines, calling for capital’s power to be openly challenged, and Poulantzas (1978: 264–5) claimed that, although such an industrial model was crucial to ‘democratic socialism’, it had to be accompanied by a political dimension.

Currently, the principal debates surround the project’s ‘trajectory’ (Upchurch et al., 2014); in the wake of the global economic crisis, mainstream sociological paradigms and social economy advocates have suggested that self-management should be used merely to construct a more ‘responsible capitalism’ and help ‘correct’ market failures by creating a new stratum of entrepreneurs (Caballero, 2004). In contrast, others understand it to be a component of an emancipatory vision that provides the only realistic path to creating a non-capitalist society (Sitrin, 2012). Neo-Marxist advocates of workers’ self-management who do not see the capture of the state as a necessary precondition for the sustainability of such cooperatives include ‘Open Marxists’ such as Holloway (2010). He argues that radical change requires creating, expanding and multiplying ‘cracks’, or quotidian moments of rebellion or autonomous spaces in capitalism. Meanwhile, ‘Autonomists’ such as De Peuter and Dyer-Witheford (2010) describe how worker cooperatives act as institutions of the ‘labour commons’ whereby surplus is distributed equitably and democratically among themselves. They claim that the radical potential of worker cooperatives can be expanded by creating networks between them and other ‘commons’ struggles. The ‘common’ is circulated, and eventually capital’s hegemony may be challenged.

Further recent debate centres upon how far work influences individual and institutional (union) identities (Foster, 2012; Marks and Thompson, 2010). For some sociologists, work has, in an historical perspective, diminished importance in shaping these identities. In so far as it relates to individuals, Foster (2012) shows that ideas of work as a simple source of self-support rather than as an identity are prominent not only in her respondents’ accounts, but also in sociological analyses. However, as we suggested earlier, developments in workers’ self-activity and experience of social relations at work demonstrate the need for a broader perspective. Positive collective experiences can help overcome workers’ alienation from their own human nature, their colleagues, their products and society more widely as Marx (1959[1844]) first argued. While work is clearly a very different experience over a century and a half after Marx wrote, his framework derives from the relationship between humans and their own needs, and offers a frame of reference that is both broader and more fundamental than the narrower ‘identity’ concept.

These contributions occur in a context in which a diluted ‘social enterprise’ paradigm has been widely propagated. Such notions take a range of forms, but have been defined as companies that apply commercial strategies to improve human and environmental well-being. They may be run on for- or non-profit bases and take the form of ‘mutuals’, ‘social businesses’ or charities (Ridley-Duff and Bull, 2011). The emphasis on this paradigm has marginalised the more radical strand and rendered it more important to examine the lived experience of the WRC experiment.

## Operationalising Halford and Strangleman’s Themes

In the opening article of their 2009 *Sociology* special issue, the guest editors described how the study of work had increasingly been shaped by post-modernism and that it had become ‘disembedded from wider social theory’ and mainstream sociology (Halford and Strangleman, 2009: 812). They appealed for research supporting a reconnection that strengthens and ‘revives’ the fragmented discipline, setting out four core themes through which this attempt could be channelled:

- ‘Control, identity and orientation to work’, with links between work and ‘non-work spaces’.
- The moral economy and class, linking individual experiences and neoliberalism.
- Industrial change, capital mobility and the meaning of work.
- New social movements and their embeddedness in communities.

The current crisis provides an opportunity to reconstruct links between the sociology of work and sociology more widely. Marxist theory – with its strengths in the analysis of crises and emancipatory responses to them – should be part of the latter. Using the analytical lens provided by these four themes, we examine the viability of Argentina’s WRCs. In finding that they offer transformatory potential as a sustainable alternative production model that fosters new non-capitalist subjectivities among workers involved, we provide a pathway that indeed reweaves the study of work into the fabric of wider social theory. This should also help regenerate industrial relations, a field centred on countries where labour movements have been relatively quiescent.

An evaluation of whether the WRCs’ aims have been maintained in practice is conducted with reference to indicators of each of the four themes (see Table 1). Evidence is then triangulated from three sources.

**Table 1.** Research themes, indicators and sources.

Themes suggested by Halford and Strangleman	Indicators of WRC viability	Source/Method
‘Control, identity and orientation to work’, with links between work and ‘non-work spaces’	Worker control over production (participation in direct democracy and collective decision-making)	PFA survey
	Collective identity and interests	WRC case studies
	Workplace as a multifunctional site Frontiers of control	WRC case studies
The moral economy and class, linking individual experiences and ‘neoliberalism’	Social goals (job creation, wages, public goods provision) not profit	PFA survey
	Wage equality	WRC case studies
	Absence of ‘exploitation’	
	Non-capitalist subjectivity and class consciousness	
Industrial change, capital mobility and meaning of work	Work as alienation or self-realisation	WRC case studies
	Work understood as a coerced or free act	WRC case studies
New social movements and their embeddedness in communities	WRCs emancipatory or reformist agenda	Document analysis
	Reproducing or rupturing capitalist social relations	PFA survey/ literature
	Relationship with the state (cooptation or autonomy)	PFA survey/ document analysis

First, we interpret data from the University of Buenos Aires’ Open Faculty Programme’s (Programa Facultad Abierta – PFA) 2010 report; *The Recovered Companies in Argentina*. Providing survey analysis of the panorama of Argentina’s WRCs, it quantitatively and qualitatively assesses their development. Establishing the survey universe was necessarily conducted using subjective criteria; if the survey authors deemed the company to have once been privately owned but now run ‘collectively’ by workers following a takeover, then it was included. With 85 WRCs having been visited and having provided the answers to 121 interview questions, the survey is the most extensive and reliable to date; although it was not perfectly representative of the national movement as WRCs in Argentina’s interior were slightly under-represented (PFA, 2010: 7).

This is the third such report since 2002, allowing WRCs’ evolution to be explored in detail. We use it to evaluate a) the process and nature of internal decision-making and level and quality of workers’ participation, b) the degree of equal distribution of revenue among the workforce, c) how far surpluses are invested in generating new sources of work or social projects, d) the extent to which workers experience exploitation or self-exploitation (Heller, 2004), and e) their business and trading practices. The latter is especially useful for understanding whether or not WRCs have been completely subsumed by market logic.

Second, case study evidence is taken from a range of important ethnographic studies that have been published in recent years. These combine 100 in-depth interviews conducted with workers and include WRCs of varying sizes and industries and with different degrees of worker participation in decision-taking and pay structures. Featured WRCs are located in Buenos Aires Province, Greater Buenos Aires or its Metropolitan District – home to 71 per cent of these companies (PFA, 2010: 9).

Scrutinising these sources will help ascertain how worker subjectivities have been re-moulded through the experience of participation in WRCs. Particular attention is paid to their understandings of the meaning of work, collective and individual identities, their sense of control over production and class consciousness. The ethics and vigour of the model can thus be explored based upon how far workers have constructed alternative meanings of work that diverge from dominant neoliberal discourses.

Third, documentary analysis of several of the WRC confederations’ founding statements and government policy papers between 2003 and 2013 is also used. These enable a more grounded understanding of the stated goals and values of the WRC movement, allowing us to assess their success in accomplishing their ideological aims and in effecting practical influence on the broader labour movement.

Triangulation of PFA’s statistical data, the analysis of direct quotations (rather than authors’ interpretations of them) from WRC participants in the five case study sources, and documentary analysis permits findings to be corroborated, whilst simultaneously allowing any significant inconsistencies between sources to be highlighted. Although this approach has limitations, the method is designed to mitigate the reality that almost all WRC literature is by authors who are ideologically sympathetic to the movement.

Table 1 summarises our method. In the first column, Halford and Strangleman’s (2009) themes appear; in column two the indicators used to explore them are highlighted; our method(s) are noted in the final column.

## WRCs: Origins and Development

The Argentine experience of self-management emerged during a social and economic depression linked to a wider crisis of élite political legitimacy. The government committed the largest sovereign debt default in world history ($93 billion); GDP plummeted by 20 per cent between 1999 and 2002 (ECLAC, 2011) and one in four citizens were left unemployed (INDEC, 2003). The crisis followed a decade of IMF-induced neoliberal reforms and austerity programmes that reduced labour and social security rights.

Meanwhile, the electorate manifested deep disillusionment with established parties, politicians and the institutions of representative democracy. In response to widespread rioting in December 2001, the government’s declaration of a State of Siege sparked enormous popular *cacerolazo* pots and pans protests. However, rather than simply protest *against* the failures of neoliberalism and representative democracy, citizens actively engaged in a variety of collective actions inspired by ideas of social transformation and autonomy (Flores, 2002; Ozarow, 2014) and which practically rehearsed different ways of organising society that moved *beyond* the existing paradigm (Holloway, 2010). These included the creation of popular and neighbourhood assemblies, the establishment of thousands of barter clubs (used by millions of Argentines), replacing money as the means of exchange, and the resurgence of the *piqueteros* – unemployed workers’ movements (Movimiento de Trabajadores Desocupados, MTD) which transcended demands for food and jobs by actively creating dignified sources of work.

The formation of WRCs represented an attempt to recover jobs in a context of high unemployment and few welfare safety nets following President Menem’s neoliberal reforms in the 1990s (Monteagudo, 2008), and to overcome the injustice of being owed substantial unpaid wages (Fernández et al., 2008).^1^ However, they also exhibited a sharp political edge, as they occurred within the context of this wider rebellion (Dinerstein, 2007). Despite Argentina’s more recent return to troubled economic times, WRCs have subsequently thrived during a decade of almost uninterrupted high growth since 2003 (IMF, 2013), together with comparatively low unemployment (ECLAC, 2011), and a return to political normality.

WRCs emerged in companies where production had ceased due to bankruptcy or the enterprise entering administration. After owners abandoned the premises, workers conducted ‘tomas’ (‘takeovers’) and decisions were taken to recommence production. Where the business had become insolvent, a physical takeover of the plant was necessary because Argentine bankruptcy law requires company assets to be auctioned off immediately, to pay creditors. The removal of machinery was therefore impeded by physical occupation (as in half of pre-2004 cases), or by blockading workplace entrances.

Argentina’s WRCs are overwhelmingly concentrated in manufacturing although a third operate in services. They have an 83 per cent male demographic (PFA, 2010: 45) and, although women benefit from certain institutionalised WRC policies (for example, the enforcement of equal pay), the degree of political leadership that they undertake internally is related to their prior union experience, and patriarchal attitudes still permeate the movement (Di Marco and Moro, 2004). This constitutes a clear equity issue.

The industries involved have strong international traditions of collective organisation; 87 per cent of these workplaces were previously unionised and there is a higher incidence of WRCs in regions with stronger histories of labour struggles and greater contemporary union presence (PFA, 2010: 59). Recovery processes acted as collective learning experiences that built on this past. Rebón (2004) suggests that on initially occupying the workplace, longer-term ‘recovery’ was not always the intended goal and that if an early compromise offer had been made by the owner, workers would probably have agreed. Instead, workers’ consciousness developed as protracted processes unfolded and the idea of continuing production without a boss emerged. In 90 per cent of WRCs, following the plant occupation, the ‘self-management’ idea was proposed to workers by outsiders, usually representatives from the recovered companies umbrella movements (described below), left-wing parties or unions. After self-management (*autogestion*) is agreed to, this external ‘promoter’ usually remains involved.

At least 87 per cent of WRCs established in the early 2000s survive today. Their numbers have grown from 161 in 2004 to 309 in 2013, doubling the number of workers involved, to 13,400 (PFA, 2013). Where self-managed companies have fully consolidated operations, workers receive higher remuneration than in equivalent traditionally run companies (Magnani, 2011). This is impressive since workers often inherit their factories without electricity, with broken machinery and assuming previous owners’ crippling business debts.

Yet their legal status is often problematic. Whilst legal recognition is usually gained by acquiring ‘cooperative’ status, few courts have recognised workers as legal proprietors of the premises they produce in. A decade after their emergence, only 12 per cent of WRCs had been granted permanent expropriation. This legal precariousness has resulted in lengthy and costly legal battles, severely hampering their development (PFA, 2010: 24–5). WRCs also clearly have to compete with other firms in product and service markets, and are thus subject to market forces, albeit without external owners to remunerate.

State support has greatly assisted their survival. Eighty-five per cent have received national, provincial or municipal subsidies (PFA, 2010: 70). In 2011, the government also started to encourage the formation of new cooperatives through Bankruptcy Law 26,648, which prioritises worker acquisition rights when businesses close down. To prevent the WRCs from spearheading an autonomous solidarity economy ‘from below’ (seen as a serious threat in the immediate aftermath of the crisis), a series of state-sponsored social programmes were launched between 2003 and 2005. These created thousands of worker cooperatives that were considerably less politicised, and subject to far tighter state control than the WRCs. Currently Argentina boasts 16,000 such businesses which provide 300,000 jobs and account for 10 per cent of GDP (Silveira, 2011). Such corporatist practices revive a favoured response of Peronist governments to restrain the more radical and autonomous elements of working-class mobilisation (James, 1993).

Despite some positive features, including according workers greater dignity increasing their voice in the production process and aiding social inclusion by providing jobs for the structurally poor, these government-created cooperatives operate in accordance with traditional capitalist principles. Large wage disparities are common and decisions are delegated to an administrative council rather than a workers’ assembly. This limits worker democratic participation, and they have become the tools of political clientelism (Colina and Giordano, 2011). These policies, together with the granting of state subsidies to WRCs, have depoliticised workers’ stated aims of autonomy, self-management and solidarity and have partially demobilised the movement as a political force (Dinerstein, 2007).

WRCs have nevertheless fought to maintain themselves as a distinctive form of cooperative. Solidarity with other social movements has played a vital role in the consolidation process and in shielding WRCs from adopting capitalist forms of organisational behaviour (Flores, 2002). Material support from other self-managed enterprises was received by 68 per cent of WRCs during their ‘recovery’, rising to 82 per cent in the longer term. Although only 14 per cent of their clients are sister WRCs (PFA, 2010: 36), given that there are only 309 such entities in the whole of Argentina, this demonstrates a vastly disproportionate preference towards doing business with them. Recently, WRCs and cooperatives have begun to engage in sectoral vertical integration strategies by which different worker-owned companies in particular industries share resources, markets and expertise in order to eliminate the pressures of capitalist competition (Giuffrida, 2011). Building the solidarity economy and producing for social ends are expressions of how WRCs aid the circulation of the ‘commons’ and, in classical Marxist terms, contribute to workers overcoming alienation from their products and society. Thus, while market, legal and political pressures on the WRCs have been severe, they have sought to maintain their principles of equity and self-management and to extend the links between them.

## Evaluating Argentine Workers’ Self-management

Broad assessments of the experience differ considerably. Whilst English-language material remains sparse, discussion within this literature has focused on whether the WRCs help to reproduce capitalism or represent a break with its logic. Kabat (2011) emphasises that although they have been important in terms of workers recovering some control following a decade of neoliberal restructuring in the 1990s, they have since helped to sustain the capitalist economy, noting that the government favours facilitating worker buyouts over nationalisation. Observing labour process dynamics on the shopfloor, Atzeni and Ghigliani (2007) also argue that although worker-recoveries establish more democratic workplaces, collective decision-making is inevitably weakened by their enterprises’ need to compete in the market as it leads to a centralisation of workplace power.

Yet Sitrin (2012) provides a more optimistic assessment, proposing that, although in the capitalist economy the spheres of ‘production’ and the ‘satisfaction of needs’ are separate, under workers’ self-management they are deemed to be indivisible because the business goals are to reproduce life (not capital). Therefore WRCs’ accomplishments should not be measured purely by commercial indicators but first, by their capacity to establish new forms of sustainable economic activity that correspond with the non-capitalist principles of solidarity, and second, whether workers produce with dignity while creating new subjectivities. Their very existence may, as Holloway (2010) advocates, demonstrate the beginning of cracks in the capitalist mode of production.

In the following sub-sections we continue this debate by appraising WRC vitality, with specific reference to the four key themes identified above.

### Control, Identity and Orientation to Work: Links between Work and ‘Non-work’ Spaces

Evidence suggests that WRC workers enjoy greater participation in decision-making, despite some problems. Two organisational structures have emerged: ‘horizontal’ and ‘vertical’. Horizontal structures are used by 70 per cent of companies and involve either managerial tasks being divided equally among all workers, or each individual assuming a broader managerial role for a short period. Despite little or no experience, or training in performing them, worker-managers have successfully replaced specialised manager roles. Some 88 per cent of WRCs regularly hold workers’ assemblies to take decisions. Yet, although many WRCs adopt an administrative council to coordinate their daily operational affairs, just 8 per cent fall into the second (vertical leadership) category and employ them as their principal decision-making instrument. In such cases, workers’ assemblies meet only once a year and the same individuals have tended to remain in ‘managerial’ positions (PFA, 2010).

Thus in the overwhelming majority of cases, the principle of workers’ democracy remains institutionally embodied. The principle tends to be most clearly in evidence where the enterprise has: a) fewer personnel (because greater intimacy induces more direct interactions); b) lower levels of internal stratification existed prior to self-management (because pre-existing organisational cultures persist); and c) experienced a high intensity of physical or legal conflict while ‘recovering’ the enterprise (because of the bonds developed among workers during the process) (Rebón, 2004). The practical application of workers’ democracy cannot accurately be measured purely by the existence of such structures and policies, but its presence in the WRCs is supported by the case study analysis conducted below.

WRC self-awareness has been honed through dialogue with unions, which at least initially regarded their workers either as ‘managers’ or as engaging in self-exploitation (Heller, 2004). Union suspicion emanates from their memories of the 1990s when companies made workers redundant and offered them the possibility of forming ‘cooperatives’ which managers then asserted control over, effectively treating them as outsourced workers (Antivero et al., 2012: 25). Although some regional branches of sectoral unions support WRCs, many local General Workers’ Congress (CGT)^2^ branches withdrew their workers’ union memberships on the grounds that they are not salaried employees (PFA, 2010: 87). Relations with unions have recently improved, perhaps because the latter have become more aware of the issues involved in shunning a popular movement. Union support was received by 34 per cent of worker-recovered companies in 2004, but the figure has risen to 64 per cent among those established since then, reflecting this change in union attitudes (PFA, 2010: 21).

With respect to the links between work and non-work spaces, workers reported that before their workplace’s ‘recovery’, they barely knew their colleagues due to strict regulations prohibiting them from discussing non work-related issues or from circulating within the workplace. This fragmentation of workers and their alienation from each other changed radically under self-management. Such exchanges were no longer penalised, and the introduction of multitasking – whereby workers would eat, cook, clean and take managerial decisions together – meant they quickly became more acquainted with others’ personalities, tribulations and dreams (Fernández et al., 2008). This engendered a new sense of community, openness and togetherness. Under workers’ self-management, the workplace has come to ‘feel like home’ (Coraggio and Arroyo, 2009: 148). In some instances, workers are permitted to use the factories or offices to book social meetings or host cultural activities, and parents may bring their children into the workplace. More generally, the factory floor has become a multifunctional site which represents a space of collective interaction and a locus of social production (Bialakowsky et al., 2005).

Some authors raise legitimate concerns that the proliferation of ‘flexible working’ within traditional forms of enterprise in the last 20 years means that work has ‘invaded’ non-work spaces (Olson-Buchanan and Boswell, 2006) to the detriment of the quality of friendships and family life (Chesley, 2005). Yet the studies examined in this research (Bialakowsky et al., 2005; Coraggio and Arroyo, 2009) suggest that in the WRCs’ case this ‘blurring’ has had the opposite effects on relationships, well-being and productivity. Whilst this paradox may seem anomalous, positive outcomes in the latter case can be explained by differences in the degree of workplace control. In WRCs, workers remain in charge without being coerced into taking such choices and, as owner-managers, also directly benefit economically. Workspaces have become sites of autonomy and self-realisation.

However, it was also apparent that in the 8 per cent of all WRCs which operated under the command of an administrative council, or where the old enterprise’s middle-managers retained their jobs, greater internal tensions and suspicion among workers were evident. Those enterprises with presidents or elected political representatives were more susceptible to divisions due to internal politics or the formation of competing interest groups that try to influence workers’ assembly meetings (Monteagudo, 2008: 197).

### Moral Economy and Class

The WRCs’ social goals have been largely upheld. They have overwhelmingly prioritised maintaining and creating sources of dignified work, higher wages or social projects for local communities over profit maximisation. In recent years 77 per cent of WRCs added to their workforce, with aggregate net WRC employment growth of 14 per cent (PFA, 2010). Although a large number of traditionally organised factories and other enterprises carried out redundancies during Argentina’s 2009 slowdown, WRCs practised work-sharing and other alternatives to redundancy by cutting working hours, reducing production or lowering their salaries. Surplus is viewed by worker-managers as their product, to be distributed equitably among those who created it, consistent with the idea of ‘the commons’ (De Peuter and Dyer-Witheford, 2010: 37).

Other indicators used to measure WRCs’ maintenance of equity from this perspective are remuneration and ‘self-exploitation’. The majority of WRCs have retained a degree of commitment to the equity principle: 56 per cent abide by a policy of equal pay and 67 per cent have the same length of working day for all. Where differences exist, they are marginal and symbolic, often expressing reward for individuals temporarily undertaking a brief fixed term of managerial office or acting as a political representative for their company. Among those cases where equal pay is not applied, only 6 per cent of companies had a wage structure with a difference of 75 per cent or more between the highest and lowest paid worker; the difference was less than 25 per cent in over half of cases (PFA, 2010: 56). Heller’s (2004) argument that WRC workers are engaged in ‘self-exploitation’ is at least questionable since: a) in fully consolidated WRCs, their earnings were higher than in comparable firms; b) jobs were secure; c) workers generally controlled their own labour process; d) wage inequality was either absent or nominal and temporary; and e) most firms rotated managerial tasks. Inequities are largely explained by varying job characteristics and responsibilities, a principle widely accepted among workers themselves (Nergaard et al., 2009).

WRC workers have dualistic comprehensions of their social reality. They understand that their enterprise is part of a movement that seeks to construct a distinctive productive logic on the one hand, yet which must operate in the market for its *organisational* survival (Bialakowsky et al., 2005) on the other. Monteagudo (2008: 194–5) argues that these new subjectivities did not evolve into classic anti-capitalist class consciousness because workers’ actions originated from economic necessity; they cannot be schooled in anti-capitalist political ideology simply from having participated in *autogestion*.

### Industrial Change and the Meaning of Work

Modern capitalist industrial relations establish two strongly differentiated subjectivities: owner-manager and (work-dependent) waged-labourer, with the latter compelled to accept a subordinate role (Fernández et al., 2008). Workers’ understandings of their position are perpetually re-interpreted, but occasionally the cycle is interrupted by a ‘crisis or pause in discourse’ (Gibson-Graham, 2006). Monteagudo (2008) argues that the 2001–2 crisis was so profound that citizens and workers re-evaluated their roles in society and the workplace. Several studies (Bialakowsky et al., 2005; Coraggio and Arroyo, 2009; Fernández et al., 2008; Monteagudo, 2008) found that subjectivities were altered and that across all cases, work has become synonymous with the recovery of dignity, self-esteem and self-realisation.

Moreover, work has been re-interpreted by those involved as a ‘free’ rather than as a ‘coerced’ act. Workers also repudiated the paternalistic relationships they had experienced with former employers. From having believed that they had been ‘looked after’ before the takeover, they came to see those perceptions as lamentable (Bialakowsky et al., 2005; Coraggio and Arroyo, 2009). Workers described how they developed a new awareness that their treatment by owner-managers had been ‘degrading’, whilst prior subjectivities by which they accepted having to provide their labour in exchange for pay *under threat of dismissal*, were now denounced as ‘humiliating’ (Coraggio and Arroyo, 2009: 143). Many explained that they were now being paid ‘what they deserved’ in the WRCs. Where companies were still in the early post-takeover ‘consolidation’ phase, even if they were not yet earning what they felt they deserved, work had become ‘enjoyable’, because they felt empowered to manage their working environment (Bialakowsky et al., 2005).

Workers report taking greater pride in their work and performing their tasks more conscientiously because they recognise that they now produce for themselves (and each other). They have noticed their co-workers doing the same; thus a new consciousness of collective responsibility has begun to replace the ‘despotic cooperation’ (Marx, 1967[1867]) that preceded it. They have come to agree that it is in their collective interests to monitor each others’ work practices (Bialakowsky et al., 2005). Furthermore, the end of competition for promotion fosters a more collaborative culture (Coraggio and Arroyo, 2009: 147).

A minority of workers had not come to terms with the fact that they were now working for themselves and no longer had to simply take orders from above (Fernández et al., 2008; Monteagudo, 2008). They reject sacrifices in the collective self-interest and perceive the potential contradictions indicated above. Although, generally, workers are incentivised by collective self-interest, there has therefore been an ‘uneven and unequal development’ of new subjectivities (Monteagudo, 2008: 197), which has sometimes provoked tensions.

### New Social Movements and their Embeddedness in Communities

We now analyse the extent to which the WRCs can be described as a ‘social movement’ with a shared vision for labour, and examine whether they purport to exist harmoniously within a capitalist framework or advance a transformative agenda. This necessitates an understanding of how the movement relates to the state (Upchurch et al., 2014) and of associated fissures in the movement.

Dinerstein (2007) observes how two distinct political visions have emerged. The first perceives workplace recoveries as a tool for a working-class revolutionary strategy and demands the elimination of exploitation and capitalist social relations. Such positions are espoused by the National Movement of Recovered Companies (MNER), WRCs such as the Brukman textile factory and the political parties of the organised Left. This tendency demands that the WRCs are brought into public ownership as this, they claim, is the only way they can be protected from market assimilation. The second, alternative ideal held by the majority of WRCs is that workers’ self-management is an end in itself and WRC autonomy is acknowledged to be limited by market forces. Rather than capture the state, these seek to create a parallel ‘solidarity economy’.

Regardless of these strategic disagreements, the movement is largely united in its emancipatory ideals. Seventy-nine per cent of WRCs are affiliated to national federations of self-managed companies that at least rhetorically seek the establishment of an alternative society with a production model based on non-capitalist principles, alongside the explicit demand for an end to exploitative labour relations. Just 16 per cent belong to federations that do not explicitly seek a long-term challenge to the economic status quo (PFA, 2010: 77).

Nevertheless, with the WRCs split into at least six separate socio-political organisations, the movement is politically highly fragmented. This derives partly from tactical disagreements. Thus the MNER favours confrontational street mobilisations to pressure the government, whereas the National Movement of Worker-Recovered Factories (MNFRT) prefers government dialogue and pursuit of the legal path (Ranis, 2010: 84). Division also derives from differences in how they interact with the state, which most regard with suspicion. Workers at some WRCs identified President Nestor Kirchner (2003–7) and since then his wife Cristina’s populist governments as allies – albeit one that only supports depoliticised strands of workers’ self-management as part of their National-Popular project. The Argentine Federation of Cooperative and Self-Managed Workers (FACTA) fully endorses such perspectives, yet, at the other end of the spectrum, the MNER is much more sceptical of the state’s activities, whilst a range of intermediate positions are adopted by different umbrella organisations.

Now that the transformative, albeit gradualist *intent* of the vast majority of WRCs has been established, the issue of whether they are producing a rupture in capitalist social relations (Holloway, 2010) or are merely reproducing them may be re-visited. On the one hand, workers’ self-management has undeniably helped buttress the market economy and in this sense sustains capitalism. WRCs’ main suppliers are large, capitalist enterprises (used by 47%), SMEs (46%) and sector-specific monopoly firms (33%). A much lower proportion of business is conducted with social enterprises (2.5%) or other WRCs (16%) (PFA, 2010: 35–6). On the other hand, individual enterprises have sought to create non-capitalist spaces from which to launch autonomous economic empowerment strat-egies. Thus the Graphics Network brings together 15 cooperatives and WRCs at different points in the printing and design industry’s supply chain. The aim of this vertical integration strategy is not only to maximise production levels by working in partnership but, crucially, to cultivate a counter-hegemonic project (Giuffrida, 2011). Similar examples exist in the health and hospitality sectors. Despite participation in the solidarity economy, WRCs are constrained by the laws of the market. The process of workplace recovery is therefore undoubtedly being assimilated to some extent into élite perspectives. This has occurred first through the WRCs’ inevitable acceptance of state subsidies and technical support; second, due to being cajoled into normalisation by liberal democracy’s legal framework; third, by the need to access credit from the banks and financial institutions; and fourth, due to the need to trade. Nevertheless, vertical integration projects counter the logic of capital, presenting a viable if small-scale alternative model. In this limited sense, the ‘circuit of capital’ is being broken (Marx, 1967[1867]).

Implicitly recognising their prior conditions of alienation, WRC workers realise that they do not produce in conditions of social isolation and that nurturing strong relationships with local communities must become central to their project. Thus, the FaSinPat factory in Neuquén province hired workers from the local MTD, donates its tiles to nearby community centres and hospitals and built a health clinic in a poor local neighbourhood. In return, local people mobilised to defend the factory from successive eviction attempts, and became new clients themselves (Magnani, 2011). Another WRC, the IMPA metalwork and plastics factory in Buenos Aires, established a cultural centre that stages artistic events, training and workshops. Alongside the MTDs, in 2004 it also created *bachilleratos populares* – popular educational programmes – and a Workers’ University which rejects the ‘false neutrality’ of the mainstream education system, aiming instead to promote alternative forms of social organisation.^3^ Over half (57%) of Argentina’s WRCs organise solidarity activities such as those described (PFA, 2010: 79).

## Conclusion

We initially asked to what degree Argentina’s WRCs have preserved their central values of equity and worker self-management. Survival is a clear precondition. Despite enormous legal, logistical and commercial pressures, tensions with unions and attempts at cooptation from the state, the WRCs have almost without exception survived and flourished since 2001. This is consistent with European experience, where workers’ cooperatives were up to three times more likely to survive the economic crisis in Italy between 2007 and 2010 than other forms of enterprise, and 50 per cent more able to do so in France in 2012 (CECOP, 2013: 11–12). The model is clearly viable.

They have also succeeded in maintaining their central values. Managerial decisions are taken and applied within a framework of non-capitalist ideals. We therefore rejected the ‘self-exploitation’ thesis as simplistic and reductive of the central trade-offs between involvement and episodic sacrifice, and between the long and short term. All workers’ organisations must simultaneously balance accommodation and resistance to capitalism across a range of issues, but fundamental qualitative gains have been maintained. WRCs more closely resemble workplace communities, or Weberian *Gemeinschaften*, than traditional capitalistic firms. Whatever compromises have been made, WRCs are not run on contemporary management principles. An alternative moral economy to that of neoliberalism is asserted within and beyond them. In Marxist terms, it appears that workers’ alienation from themselves, from other workers, their products and society is at least partially overcome.

Limitations are evident. Equity has been largely maintained in remuneration terms but gender equity is more problematic. The quality of democracy is clearly variable. Moreover, both the numerical dominance of male workers and blurred boundaries between ‘work’ and ‘home’ pose the danger that patriarchal patterns of behaviour extend beyond social units such as the family and household into the workplace, leaving women often playing ‘supportive’ roles to their male counterparts (Di Marco and Moro, 2004).

Nevertheless, the broader socio-political repercussions of the WRCs’ persistence, despite their imperfect achievements, are considerable and belie the movement’s size. Their formation was the inspiration behind and catalyst for the Kirchner administrations’ championing of the cooperative movement in its discourse and microeconomic policy. The cooperative sector’s growth has naturalised the idea of ‘companies without bosses’ in the popular imagination. It has (re)created a potentially powerful new instrument (Hirtz and Giacone, 2013) which is now part of Argentine labour’s repertoire of collective action. Its transnational significance and ‘resonance’ (Holloway, 2010) is also apparent, as we argued earlier.

These developments open up possibilities for the reinvigoration of sociology by reconnecting the sociology of work with the study of wider societal issues. Halford and Strangleman argued that this was required if the subject was to rediscover its broader relevance, and other researchers have also recently contended that the study of labour management internationally requires a similar broadening of its terms of reference (Delbridge et al., 2011). We have shown that all four of Halford and Strangleman’s themes are in evidence, albeit to different degrees, in the WRCs’ recent trajectories.

The experience also offers prospects for the related and increasingly embattled field of industrial relations. The original impetus behind the field owed a good deal to the concept of industrial democracy which links the workplace and workers’ lives to wider society. This initial concern has become increasingly less explicit and the field has arguably become more influenced in Britain and the USA by optimistic accounts of institutional ‘union renewal’ (McIlroy and Croucher, 2013). Even in these terms, further in-depth inquiries could analyse union activists’ interactions with WRCs. How unions react to, learn from and interact with such significant experiments will likely indicate their capacity to learn from all external organisations, especially those with transformative visions which potentially offer at least part of their earlier utopian vision.

## Notes

1. Cited by 51 per cent and 58 per cent of survey respondents respectively (PFA, 2010). Between 2004 and 2009, the proportion who stated that their aim was to recover back pay trebled.

2. Argentina’s main, Peronist-led union confederation.

3. http://impalafabrica.org.ar/
